# Transcript Long‐Read Sequencing Unveils the Molecular Complexity of a Novel *ROGDI* Splicing Variant in a Tunisian Family With Kohlschütter‐Tönz Syndrome

**DOI:** 10.1111/cge.14725

**Published:** 2025-02-24

**Authors:** Miriam Essid, Sana Karoui, Mouna Zribi, Thouraya Ben Younes, Louis Januel, Estelle Lafont, Audrey Labalme, Meriem Ben Hafsa, Go Hun Seo, Safa Khatrouch, Hela Boudabous, Amel Ben Chehida, Damien Sanlaville, Houweyda Jilani, Lamia Benjemaa, Ichraf Kraoua, Gaetan Lesca, Nicolas Chatron

**Affiliations:** ^1^ Genetics Department Hospices Civils de Lyon Lyon France; ^2^ Neuromyogene Institute, Pathology and Genetics of Neuron and Muscle, CNRS UMR 5261 INSERM U1315 Université Claude Bernard Lyon 1 Lyon France; ^3^ Faculty of Medicine of Tunis University of Tunis El Manar Tunis Tunisia; ^4^ Genetic Department LR22SP01, Mongi Slim Hospital La Marsa Tunisia; ^5^ Department of Pediatrics and Hereditary Metabolic Diseases LR12SP02, La Rabta Hospital Tunis Tunisia; ^6^ Department of Child and Adolescent Neurology LR18SP04, National Institute Mongi Ben Hmida of Neurology La Rabta Tunisia; ^7^ Division of Medical Genetics 3billion Inc. Seoul South Korea

**Keywords:** Kohlschütter‐Tönz Syndrome, long‐read sequencing, *ROGDI*, splicing variant

## Abstract

Kohlschütter‐Tönz Syndrome (KTS) is an ultra‐rare autosomal recessive disorder, characterized by a clinical triad: infantile‐onset epilepsy, global developmental delay, and amelogenesis imperfecta. KTS is caused by pathogenic variants in *ROGDI*, encoding a leucine zipper protein of unknown function. Our study characterizes a novel homozygous *ROGDI* variant (NM_024589.3:c.646‐2A>G) identified in a Tunisian family case with KTS, renal tubular acidosis, and hyperammonemia. This variant disrupts a canonical acceptor splice site (ASS) in intron 8. Reverse‐transcriptase polymerase chain reaction and targeted long‐read cDNA sequencing, identified only abnormal transcripts secondary to the *ROGDI* ASS variant in the proband. Complex splicing events were detected including exon 9 skipping, cryptic ASS activation leading to 13‐bp deletion in exon 9, and retention of intron 8 or both intron 8 and 9. These alterations were all predicted to result in nonsense mediated decay and *ROGDI* loss of function. By integrating complementary techniques, our study unveiled fundamental mechanisms underlying complex splice alterations, providing insights that may guide future therapeutic strategies in KTS.

## Graphical abstract

We characterized a novel *ROGDI* variant (NM_024589.3:c.646‐2A>G) identified in a family case of Kohlschütter‐Tönz Syndrome. This variant disrupts a canonical acceptor splice site, in intron 8. By combining RT‐PCR and targeted long‐read cDNA sequencing. cDNA sequencing, our findings demonstrated that the splicing alterations are more complex than initially predicted by computational tools.

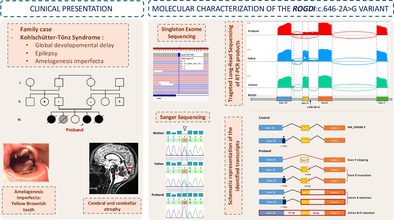

Kohlschütter‐Tönz Syndrome (KTS, MIM 226750) is an ultra‐rare autosomal recessive disorder, characterized by a clinical triad: infantile‐onset drug‐resistant epilepsy, global developmental delay (GDD), and amelogenesis imperfecta [1]. KTS, first described in 1974, was linked to biallelic loss‐of‐function (LoF) variants of *ROGDI* in 2012 [2, 3]. *ROGDI* (MIM 614574) encodes a 287‐amino acid atypical leucine zipper protein [1]. So far, only 46 patients have been reported and limited *ROGDI* splicing variants has been investigated by RT‐PCR and Sanger sequencing, leaving alternative splicing effects largely unexplored [1, 2, 4, 5]. We describe a novel *ROGDI* splicing variant investigated by RT‐PCR and targeted long‐read sequencing, revealing complex splicing outcomes.

The patient was a 10‐year‐old girl born to healthy consanguineous Tunisian parents. Two sisters with the same presentation deceased in infancy (Figure 1A). She experienced the first generalized tonic–clonic seizure at 6 months. Initial therapy based on valproic acid (VPA) was ineffective. GDD was evident from early infancy. At 9 years of age, she presented with drug‐resistant epilepsy, motor regression, feeding difficulties, and severe growth retardation. Neurological assessment demonstrated absence of language, spastic quadriplegia, and hand stereotypies. Repeated EEG‐recordings were normal. Brain MRI revealed diffuse cerebral and cerebellar atrophy (Figure 1B). The patient was dysmorphic and demonstrated amelogenesis imperfecta. She was diagnosed with distal renal tubular acidosis (dRTA) and secondary rickets. Hyperammonemia, induced by long‐term VPA therapy, was worsened by hypocarnitinemia due to renal loss and undernutrition. Ammonia levels significantly decreased after switching from VPA to levetiracetam and L‐carnitine supplementation.

**FIGURE 1:**
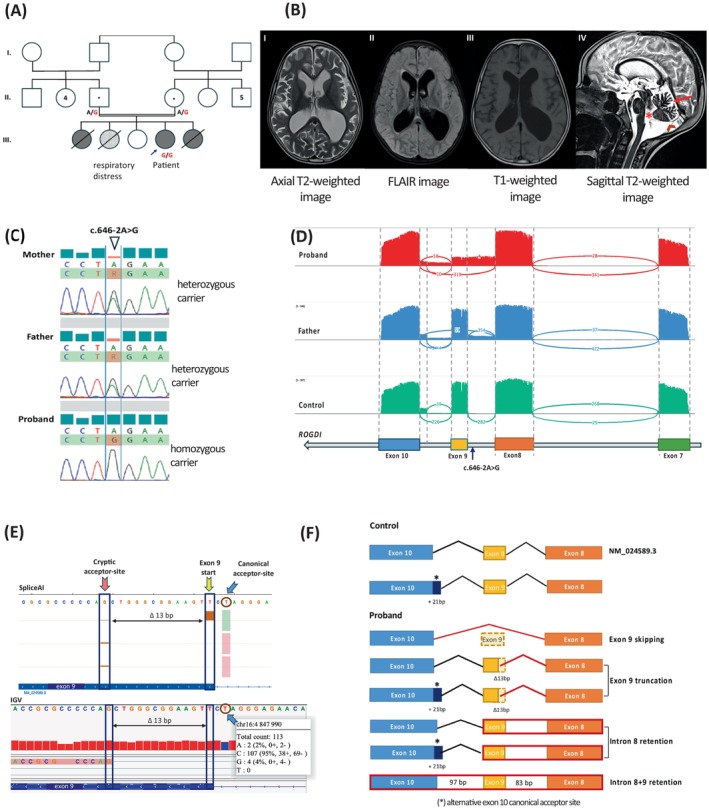
(A) Family pedigree. (B) Brain MRI images: Diffuse cortical and subcortical atrophy, cerebellar atrophy, with passive ventricular enlargement. Red arrow: Diffuse cerebellar atrophy, (*) passive enlargement of the fourth ventricle. Red arrow head: Mega cisterna magna. (C) Sanger sequencing. (D) Sashimi plots of targeted long‐read cDNA sequencing with partial schematic representation of *ROGDI*. (E) Targeted‐LRS and SpliceAI predictions. (F) Schematic representation of the identified transcripts.

Singleton exome sequencing identified a homozygous variant NM_024589.3:c.646‐2A>G in *ROGDI*, affecting the canonical acceptor splice site (ASS) in intron 8. It was detected once in gnomAD v4.1.0. The CADD phred score was 33. MaxEntScan predicted a decrease in the strength of the canonical ASS (from 9.78 to 1.83 [−81.29%]) and a cryptic acceptor site activation 15‐bp downstream (from 1.18 to 3.09 [+161.86%]). SpliceAI predictions aligned with MaxEntScan: ASS loss (Δ score: 0.88) and the same cryptic site activation (Δ score: 0.08). Both in silico tools suggested a disruption of the ASS, potentially causing skipping of the 50‐bp length exon 9 or a 13‐bp deletion in exon 9. Parents were heterozygous carriers (Figure 1C). The variant was classified as likely pathogenic (PM2_P and PVS1) according to the ACMG criteria.

RT‐PCR of lymphocyte‐derived RNA amplified the region between exon 7 and 10. Gel electrophoresis analysis of the proband revealed only abnormal bands, with a predominant band corresponding to exon 9 skipping (data no shown). Targeted long‐read sequencing analysis using Oxford Nanopore Technologies further characterized the aberrant transcripts (Figure 1D): a major product lacking exon 9, and three minor products including: (i) 13‐bp deletion in exon 9 due to cryptic ASS activation (Figure 1E), (ii) retention of out‐of‐frame intron 8, and (iii) retention of both introns 8 and 9 (Figure 1F). All these transcripts lead to premature termination codons and subsequently mRNA degradation via nonsense‐mediated decay. Interestingly, only the first two effects were predicted by SpliceAI and MaxEntScan.

The patient showed classic symptoms of KTS with unusual features including hyperammonemia and dRTA without nephrocalcinosis. Furthermore, in this case, the combination of VPA and carnitine deficiency led to hyperammonemia. Given the metabolic disorders in KTS patients, VPA should be avoided.

From a genetic perspective, the patient carries a novel splicing variant, which represents the most prevalent type, accounting for 9 of the 15 reported variants [1, 4]. Our study provided additional evidence to classify the c.646‐2A>G *ROGDI* variant as pathogenic (PVS1_VS, PM2_P, and PP3). Through the integration of advanced transcriptomic analysis, our findings enlighten potential therapeutic strategies targeting splicing defects in KTS.

## Ethics Statement

Written informed consent was obtained from the patient's parents. This study was approved by the local ethical committee in Mongi Slim Hospital, La Marsa (number 43/2024).

## Conflicts of Interest

The authors declare no conflicts of interest.

## Data Availability

The data that support the findings of this study are available from the corresponding author upon reasonable request.
